# Evaluation and Measurement of Health-Related Quality of Life for Individuals with Diabetes Mellitus by Health Utilities Index Mark 3 (HUI3) System

**DOI:** 10.1100/tsw.2006.253

**Published:** 2006-11-14

**Authors:** Frank Mo, Howard Morrison, Bernard C.K. Choi, Lianne Vardy

**Affiliations:** Centre for Chronic Disease Prevention and Control, Public Health Agency of Canada, 120 Colonnade Road, Ottawa, Ont. K1A 0K9, Canada

**Keywords:** health utilities index, quality of life, diabetes, longitudinal health survey, Canada

## Abstract

The Health Utilities Index Mark 3 (HUI3) is an important indicator when measuring the health-related quality of life (HRQOL) and assessing the burden of disease, especially for chronic conditions such as diabetes mellitus (DM). The objectives of this study were to evaluate the scores of HRQOL for respondents with DM to examine associations between overall HUI3 scores and eight component attributes, and various sociodemographic and lifestyle attributes, and by doing so, provide information to improve the HRQOL of individuals with diabetes. The study was based on the Canadian National Population Survey (NPHS) longitudinal data, from 1994—1995 to 2002—2003. We evaluated overall HUI3 scores and eight attributes (vision, hearing, speech, ambulation, dexterity, emotion, cognition, and pain) between respondents with and without diabetes in relation to demographic characteristics (age, sex, lifestyle, and socioeconomic status using ANCOVA[analysis of covariance]). Awareness of diabetes appeared to affect the HRQOL of older age groups more so than younger age groups (*p* < 0.01). Diabetes also appeared to have a greater impact on males’ quality of life compared to females', and among individuals with single marital status and low socioeconomic status (*p* < 0.01). These findings add to what is known about cognitive representations and the self-regulation of diabetes as well as the relationships between cognitive representations of diabetes, HRQOL, and behavioral factors. In particular, results from this study suggest the need to address ways of reducing the burden of diabetes associated with health behaviours, and increasing the quality of life for the individuals with diabetes in Canada.

### INTRODUCTION

Health-related quality of life (HRQOL) is an important concept, not only in health, but also in health economics. It has been particularly useful in characterizing the health burden of chronic diseases, such as diabetes[1,2,3]. The concept of HRQOL and living "a good life" has become the subject of a number of scientific and political debates[4,5,6]. There is an increasing appreciation of the importance of enhancing the HRQOL for individuals with chronic diseases.

The Health Utilities Index Mark 3 (HUI3), which has been considered to be an important indicator for assessing the burden of disease especially for chronic conditions, is based on the Comprehensive Health Status Measurement System (CHSMS) and a generic multiattribute preference-based measure of health status. It is widely used as an outcome measure in clinical studies, in population health surveys, in the estimation of quality-adjusted life years, and in economic evaluations[7]. It also provides a description of an individual's overall functional health, based on overall HUI3 and eight attributes: vision, hearing, speech, ambulation (ability to get around), dexterity (use of hands and fingers), cognition (memory and thinking), emotion (feelings), and pain/discomfort[8]. The HUI3 was developed by McMaster University's Centre for Health Economics and Policy Analysis, and is derived using societal preferences from a random sample of 504 people within the boundaries of the City of Hamilton-Wentworth, Ontario, Canada[9].

The Health Utilities Index describes a system for measuring the overall health status and HRQOL of individuals, clinical groups and general populations with chronic disease. It is a member of a family of generic health status and HRQOL measures that includes the Health Utilities Index Mark 1 (HUI1), Mark 2 (HUI2), and Mark 3 (HUI3) systems, which are frequently used both in clinical and population health studies in Canada[10]. The HUI3 consists two components: (1) the health status classification system and (2) the preference-based scoring system[11,12].

The objectives of this study are to evaluate the scores of HRQOL for respondents with diabetes mellitus (DM) in order to detect possible associations between overall HUI3 scores, eight attributes, and various aspects on lifestyle and socioeconomic status, and by doing so, provide information to improve the HRQOL for individuals with diabetes.

### METHODS

Data were obtained from the National Population Health Survey (NPHS)[13], Longitudinal Data, from 1994–1995 to 2002–2003. NPHS collects information related to the health of the Canadian population and related sociodemographic information. It is composed of three components: the Households, the Health Institutions, and the North components. The Household component started in 1994–1995 and is conducted every 2 years. The first three cycles (1994–1995, 1996–1997, and 1998–1999) were both cross-sectional and longitudinal. Beginning in cycle 4 (2000–2001), the survey became strictly longitudinal (i.e., collecting health information from the same individuals ascertained in 1994–95 in each cycle). The NPHS longitudinal sample includes 17,276 persons aged 12 years and above in 1994–1995. These same persons will be interviewed every 2 years over a period of 18 years, i.e., 10 cycles. The NPHS data collection is performed using a Computed Assisted Interview (CAI) system[14]. The logical flow of the questions is programmed to reflect skip patterns associated with certain variables such as age. The computer program also takes into account the type of answer required, the allowed minimum and maximum values, online edits associated with the question, and what to do in case of item nonresponse.

The questionnaire for each cycle of NPHS is prepared in collaboration with specialists from Statistics Canada, Health Canada, Public Health Agency of Canada, provincial ministries of health, researchers from the academic fields, and other stakeholders. Every questionnaire is approved by members of the expert committees and the Advisory Committee[13].

### Target Population

The target population of the longitudinal NPHS includes household residents 12 years and above from all provinces, excluding populations on Indian Reserves, Canadian Forces Bases, and some remote areas in Québec and Ontario. There were 401 cases of DM identified in 1994–1995. From 1994–1995 to 2002–2003, there were 28 persons who died (or were lost to follow-up) and 214 new cases of diabetes.

Therefore, by 2002–2003, there were a total of 587 diagnosed cases of diabetes identified. From 1994–1995 to 2002–2003, there were 3,364 cases missed or lost to follow-up (see Fig. 1).

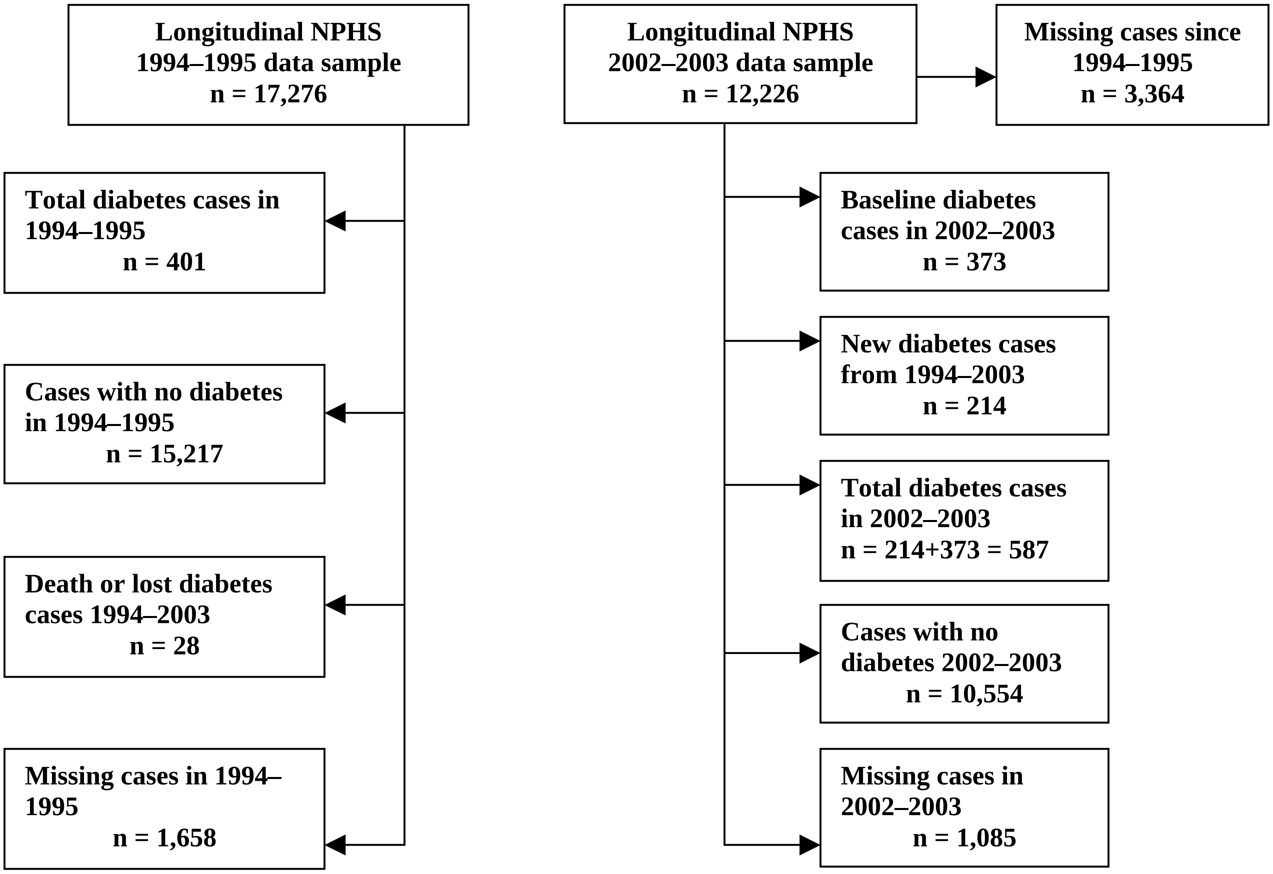

**FIGURE 1.** Study sample, NPHS Longitudinal Data of diabetes, from 1994–2003.

### Sampling

The longitudinal NPHS employed a stratified, two-stage, sample design (clusters, dwellings) based on the Labour Force Survey (LFS), which consists of a multistage, stratified sample where dwellings are selected within clusters, in all provinces except Québec. Each province was divided into three types of areas (Major Urban Centers, Urban Towns, and Rural Areas) from which separate geographic and/or socioeconomic strata were formed. In most strata, six clusters, usually Census Enumeration Areas (EAs), were selected with probability proportional to size (PPS). The sample of dwellings was obtained once listing operations in sample clusters were completed. Requirements specific to the NPHS led to small modifications to the LFS sampling strategy. The NPHS longitudinal sample is composed of 17,276 persons and is not renewed over time. The response rates for subsequent cycles are based on the 17,276 individuals who form the longitudinal panel. The cycle response rate: cycle 1 (83.6%), cycle 2 (92.8%), cycle 3 (88.2%), cycle 4 (84.8%), and cycle 5 (80.6%).

### Measures

HRQOL was measured by the HUI3 system by assigning various weights to potential health states. Overall HUI3 scores range from –0.36 for the all-worst HUI3 health state to 0.00 for dead to 1.00 for perfect health. Differences of HUI3 overall scores greater than 0.03 are considered to be clinically important[15,16,17]. Differences greater than 0.05 on a single attribute utility are considered to be important and meaningful[15,16,17]. On a population level, this type of measure is most useful for guiding health policy and for modeling what we know about health, disease, death, and their burden, especially at the national and multinational level.

### Definition of Self-Reported Type 2 Diabetes

To more accurately restrict our analysis to type 2 diabetes, we excluded responders 0–19 years of age because most cases of diabetes in those under age 20 will be type 1.

### Data Analysis

Descriptive analysis was performed to calculate the percentage of demographic characteristics of sample respondents by age, sex, lifestyle, and socioeconomic status. ANCOVA (analysis of covariance) was employed to compare the potential associations of HRQOL between overall HUI3 scores and single-attribute utility scores of each group, adjusted for age, sex, education, family income, lifestyle, and number of individuals with diabetes compared to those without. In an alternative model, we used the baseline scores of overall HUI3 and single-attribute scores from the Canadian general population as control group in 1994–1995 and in 2002–2003, and compared family income, marital status, education level, physical activity, alcohol drinking, cigarette smoking, and Body Mass Index (BMI), in order to detect the associated effects related to overall HUI3 scores and single-attribute scores within 1994–1995 and 2002–2003, and compared the differences between 1994–1995 and 2002–2003 for those with diabetes compared to those without.

## RESULTS

Overall HUI3 and eight attribute health utility scores were available for 17,276 respondents over age 12 years in 1994–1995 (see Table 1). The average age of respondents included in this study was 39.6 ± 22.7 in 1994–1995; and 44.6 ± 22.0 in 2002–2003. The 12–39 age group decreased from 31.2% in 1994–1995 to 20.5% in 2002–2003. Respondents in the 65 years and over age group increased from 19.4% in 1994–1995 to 21.8% in 2002–2003. In 2002–2003, 50.5% of respondents were female and 51.5% were single; 60.9% had a college or university degree or other form of postsecondary education, which was an increase from 52.9% in 1994–1995. The overall prevalence of diabetes in this study was 3.1 and 4.5% in 1994–1995 and 2002–2003, respectively. Of the respondents, 53.2% had a family income $50,000 or over in 1994–1995 and 70.7% in 2002–2003. Furthermore, the percentage of BMI in the category of 30.0 or greater (obese) were higher in 2002–2003 (12.2%) than in 1994–1995 (8.2%).

Overall HUI3 and eight attribute utility scores related to individuals with diabetes, adjusted by age, sex, and socioeconomic status, are shown in Table 2. The scores of HRQOL for individuals with diabetes in the ages of 40–64 and 65 years and over were statistically significantly lower than those for the younger age groups ($p < 0.01$). Male's HRQOL of diabetes was worse than female's in 1994–1995 as well as in 2002–2003 in overall HUI3 scores, and in certain single attributes (e.g., vision, emotion, hearing, pain/discomfort, and speech, $p < 0.01$).

**TABLE 1**
**Demographic Characteristics of Sample, NPHS Longitudinal Data, from 1994–2003**

| Demographics | Year 1994–1995 (n = 17,276) | Year 2002–2003 (n = 12,226) |
|---|---|---|
| Age | | |
| 12–39 | 31.2%* | 20.5%* |
| 40–64 | 49.4 | 57.7 |
| 65+ | 19.4 | 21.8 |
| Sex | | |
| Males | 49.5% | 49.5% |
| Females | 50.5 | 50.5 |
| Family income/year | | |
| Less than $30,000 | 17.3% | 9.4% |
| $30,000–49,999 | 29.5 | 20.0 |
| $50,000–79,999 | 36.6 | 35.7 |
| $80,000 or over | 16.6 | 35.0 |
| Marital status | | |
| Married | 49.8% | 48.5% |
| Single (includes divorced, separated, widowed) | 50.2 | 51.5 |
| Level of education | | |
| Less than secondary | 32.6% | 26.1% |
| Secondary | 14.5 | 13.0 |
| Other postsecondary | 23.0 | 25.4 |
| Postsecondary (college/university degree) | 29.9 | 35.5 |
| Physical activity | | |
| Active | 19.1% | 25.8% |
| Moderate | 22.6 | 27.6 |
| Inactive | 58.3 | 46.6 |
| Alcohol drinking | | |
| Yes | 51.4% | 49.9% |
| No | 48.6 | 50.1 |
| Cigarette smoking | | |
| Daily smoke | 22.9% | 18.1% |
| Occasional smoke | 5.2 | 4.9 |
| Former smoke | 30.6 | 40.4 |
| Never smoke | 41.3 | 36.6 |
| BMI | | |
| Less than 25.0 | 70.8% | 65.5% |
| 25.0–30.0 | 21.0 | 22.3 |
| 30.0+ | 8.2 | 12.2 |
| Has diabetes | | |
| Yes | 3.1% | 4.5% |
| No | 96.9 | 95.5 |

### TABLE 1 continued

| | | |
|---|---|---|
| HRQOL – Mean (SD)** | | |
| Overall HUI3 score | 0.88 (0.20) | 0.89 (0.18) |
| Vision | 0.91 (0.07) | 0.93 (0.06) |
| Hearing | 0.88 (0.11) | 0.89 (0.10) |
| Speech | 0.87 (0.08) | 0.89 (0.07) |
| Ambulation | 0.89 (0.10) | 0.90 (0.11) |
| Dexterity | 0.87 (0.12) | 0.89 (0.11) |
| Emotion | 0.91 (0.09) | 0.92 (0.09) |
| Cognition | 0.92 (0.09) | 0.93 (0.08) |
| Pain and discomfort | 0.91 (0.12) | 0.93 (0.13) |

* Percentage weighted on total Canadian population.

** Mean scores and Standard Deviation (SD) adjusted by age and sex.

### TABLE 2
## Adjusted Mean of Overall HUI3 and Single-Attribute Scores Related to Diabetes by Age and Sex, NPHS Longitudinal Data, from 1994–2003[a,b]

| **Diabetes (401 cases) 1994–1995** | **Overall** | **Vision** | **Hearing** | **Speech** | **Ambulation** | **Dexterity** | **Emotion** | **Cognition** | **Pain or Discomfort** |
|---|---|---|---|---|---|---|---|---|---|
| Control group | 0.91 | 0.98 | 0.99 | 0.99 | 0.99 | 0.99 | 0.98 | 0.97 | 0.97 |
| Age (years) | | | | | | | | | |
| 12–39 | 0.96 | 0.95 | 0.95 | 0.97 | 0.92 | 0.95 | 0.98 | 0.98 | 0.97 |
| 40–64 | 0.94 | 0.80 | 0.88 | 0.94 | 0.87 | 0.87 | 0.96 | 0.94 | 0.90 |
| 65+ | 0.80 | 0.76 | 0.67 | 0.54 | 0.64 | 0.56 | 0.90 | 0.89 | 0.85 |
| Sex | | | | | | | | | |
| Male | 0.86 | 0.91 | 0.91 | 0.95 | 0.90 | 0.98 | 0.94 | 0.96 | 0.91 |
| Female | 0.89 | 0.91 | 0.93 | 0.98 | 0.89 | 0.98 | 0.98 | 0.96 | 0.92 |
| **Diabetes (587 cases) 2002–2003** | | | | | | | | | |
| Control group | 0.92 | 0.97 | 0.99 | 0.98 | 0.99 | 0.99 | 0.98 | 0.98 | 0.99 |
| Age (years) | | | | | | | | | |
| 12–39 | 0.95 | 0.96 | 0.94 | 0.96 | 0.89 | 0.97 | 0.96 | 0.97 | 0.99 |
| 40–64 | 0.85 | 0.74 | 0.84 | 0.81 | 0.83 | 0.83 | 0.94 | 0.86 | 0.95 |
| 65+ | 0.78 | 0.73 | 0.53 | 0.40 | 0.53 | 0.42 | 0.77 | 0.77 | 0.72 |
| Sex | | | | | | | | | |
| Male | 0.86 | 0.89 | 0.90 | 0.95 | 0.90 | 0.98 | 0.92 | 0.94 | 0.91 |
| Female | 0.89 | 0.90 | 0.92 | 0.98 | 0.89 | 0.99 | 0.95 | 0.95 | 0.92 |

[a] Adjusted for marital status, level of education, family income, physical activity, BMI, cigarette smoking, and alcohol drinking.

[b] None of the entries are statistically significant ($p < 0.05$) and clinically important differences between groups compared to control group (no diabetes).

Table 3A and Table 3B show the differences between low level of education (less than secondary), low family income (less than \$30,000/year), overweight/obese (BMI equal to or greater than 30), former cigarette smokers, and the control group in the year 1994–1995 as well as in 2002–2003 in the overall HUI3 scores. In 1994–1995, both married and single individuals with diabetes were lower than the control group in all eight attribute scores, except pain/discomfort. However, these scores in 2002–2003 were all lower than the control group except married respondents in emotion and cognition attributes ($p < 0.01$).

Low level of education is an important factor affecting HRQOL in people with diabetes. The scores in the less than secondary group were significantly lower than the control group and also lower than postsecondary or other postsecondary ($p < 0.01$).

Low family income (less than \$30,000), physical inactivity, and overweight/obese in 1994–1995 and in 2002–2003 also significantly affected the HRQOL among diabetics ($p < 0.01$). However, cigarette smoking and heavy alcohol drinking were not correlated with overall HUI3 scores, except for former smokers in overall HUI3 scores, occasional smoker in cognition, and alcohol drinking in ambulation.

When we compared the overall HUI3 scores between the years of 1994–1995 and 2002–2003, we found significantly lower scores in 2002–2003 than in 1994–1995 in education less than secondary (0.86 vs. 0.82), family income less than \$30,000/year (0.88 vs. 0.84), physically active (0.99 vs. 0.93) and moderately active (0.96 vs. 0.92), overweight (BMI 25–30) (0.98 vs. 0.95) and obese (BMI >30) (0.95 vs. 0.91), and former cigarette smoker (0.97 vs. 0.92). All of these scores were greater than 0.03 and were therefore considered clinically important.

Furthermore, the differences (scores greater than 0.05) were found in the attributes in 1994–1995 more so than in 2002–2003, such as in the single married status (0.92 vs. 0.86), pain or discomfort (0.96 vs. 0.83), and the education level less than secondary in cognition (0.91 vs. 0.85). The differences were greater than 0.05 on all eight attributes in the family income less than \$30,000 in 1994–1995 than in 2002–2003. However, there were only few significant differences in the physically inactive group in emotion (0.95 vs. 0.89) and cognition (0.95 vs. 0.90). The attribute scores of overweight/obese in hearing (0.87 vs. 0.82), cognition (0.94 vs. 0.89), the score of cigarette smoking in cognition (0.99 vs. 0.94), and the scores of heavy alcohol drink in ambulation (0.97 vs. 0.92) were also lower in 2002–2003 than in 1994–1995 (see Tables 3A and 3B).

### DISCUSSION

Longitudinal population-based health survey data are useful for estimating the long-term effects on HRQOL and health status in individuals with diabetes. Diabetes is a chronic medical condition in which many factors potentially influence HRQOL and health status, some of which are disease related, but many of which are related to demographic, socioeconomics, and health care utility status[18]. Through measuring the health status of a cohort through time, we used the National Population Health Survey (NPHS), which collected longitudinal data from 1994–1995 to 2002–2003 in Canada[13].

We found that HRQOL in people with diabetes was affected by age, gender, lifestyle, and socioeconomic status. The greatest impact was found on HRQOL in the 65 years and over age group, perhaps because of a longer duration of damage caused by diabetes. HRQOL for the 65+ age group in this study was significantly worse than the 12–39 age groups in overall HUI3 scores and in the eight attributes (–0.03 to –0.56, $p < 0.01$). We also found differences in the overall HUI3 scores between 1994–1995 and 2002–2003. Significantly lower scores in 2002–2003 than in 1994–1995 were found in the level of education less than secondary, family income less than \$30,000/year, physically inactive and moderate, individuals overweight or obese, and former cigarette smokers. All of these score differences were greater than 0.03 and were considered clinically important[15,16,17]. Furthermore, differences were greater than 0.05 in the scores of some attributes in 1994–1995 as compared to 2002–2003, such as the single married status, in pain or discomfort, and education level less than secondary in cognition. The differences of scores were greater than 0.05 on all eight attributes in the family income less than \$30,000 in 1994–1995

**TABLE 3A**
**Adjusted Mean of Overall HUI3 and Single-Attribute Scores Related to Diabetes, NPHS Longitudinal Data, from 1994–2003[a,b]**

| Diabetes (401 cases) 1994–1995 | Overall | Vision | Hearing | Speech | Ambulation | Dexterity | Emotion | Cognition | Pain or Discomfort |
|---|---|---|---|---|---|---|---|---|---|
| Control group | 0.91 | 0.98 | 0.99 | 0.99 | 0.99 | 0.99 | 0.98 | 0.97 | 0.97 |
| Marital status | | | | | | | | | |
| Married | 0.98 | 0.86 | 0.86 | 0.79 | 0.81 | 0.80 | 0.89 | 0.92 | 0.96 |
| Single | 0.97 | 0.86 | 0.86 | 0.78 | 0.81 | 0.80 | 0.86 | **0.92** | **0.96** |
| Level of education | | | | | | | | | |
| Less than secondary | **0.86** | 0.83 | 0.88 | 0.86 | 0.87 | 0.87 | 0.91 | **0.91** | 0.90 |
| Secondary | 0.94 | 0.94 | 0.92 | 0.88 | 0.91 | 0.88 | 0.97 | 0.96 | 0.95 |
| Other secondary | 0.97 | 0.97 | 0.97 | 0.97 | 0.97 | 0.97 | 0.98 | 0.95 | 0.98 |
| Postsecondary | 0.99 | 0.97 | 0.98 | 0.99 | 0.98 | 0.98 | 0.99 | 0.99 | 0.99 |
| Family income/year | | | | | | | | | |
| Less than $30,000 | **0.88** | 0.93 | **0.95** | **0.95** | **0.94** | **0.97** | **0.93** | **0.95** | **0.90** |
| $30,000–49,999 | **0.92** | 0.93 | **0.97** | **0.97** | **0.93** | **0.97** | 0.94 | 0.95 | 0.93 |
| $50,000–79,999 | 0.94 | 0.98 | 0.97 | 0.97 | 0.97 | 0.98 | 0.96 | 0.97 | 0.96 |
| $80,000 or over | 0.96 | 0.99 | 0.98 | 0.98 | 0.98 | 0.98 | 0.96 | 0.98 | 0.97 |
| Physical activity | | | | | | | | | |
| Active | **0.99** | 0.98 | 0.98 | 0.97 | 0.99 | 0.98 | 0.99 | 0.99 | 0.97 |
| Moderate | **0.96** | 0.96 | 0.91 | 0.89 | 0.89 | 0.90 | **0.96** | 0.98 | 0.95 |
| Inactive | 0.89 | 0.91 | 0.86 | 0.80 | 0.86 | 0.81 | **0.95** | **0.95** | 0.87 |
| BMI | | | | | | | | | |
| Less than 25.0 | 0.99 | 0.97 | 0.98 | 0.97 | 0.97 | 0.98 | 0.99 | 0.97 | 0.97 |
| 25.0–30.0 | **0.98** | 0.93 | 0.89 | 0.88 | 0.88 | 0.88 | 0.96 | **0.95** | 0.94 |
| 30.0+ | **0.95** | 0.87 | **0.87** | 0.85 | 0.85 | 0.85 | 0.95 | **0.94** | 0.92 |
| Cigarette smoking status | | | | | | | | | |
| Daily smoke | 0.93 | 0.88 | 0.87 | 0.84 | 0.87 | 0.86 | 0.92 | 0.92 | 0.90 |
| Occasional smoke | 0.98 | 0.90 | 0.92 | 0.88 | 0.90 | 0.88 | 0.93 | **0.99** | 0.96 |
| Former smoke | **0.97** | 0.94 | 0.92 | 0.90 | 0.91 | 0.90 | 0.93 | 0.98 | 0.97 |
| Never smoke | 0.99 | 0.98 | 0.98 | 0.99 | 0.97 | 0.98 | 0.99 | 0.99 | 0.98 |
| Alcohol drinking | | | | | | | | | |
| Yes | 0.86 | 0.93 | 0.95 | 0.93 | **0.97** | 0.94 | 0.97 | 0.97 | 0.93 |
| No | 0.97 | 0.97 | 0.97 | 0.98 | 0.99 | 0.97 | 0.99 | 1.00 | 0.97 |

[a] Adjusted for age and sex.

[b] Bold entries are statistically significant difference (0.03 for overall scores and 0.05 for single-attribute scores) and clinically important differences between 1994–1995 and 2002–2003.

**TABLE 3B**
**Adjusted Mean of Overall HUI3 and Single-Attribute Scores Related to Diabetes, NPHS Longitudinal Data, from 1994–2003[a,b]**

| Diabetes (587 cases) 2002–2003 | Overall | Vision | Hearing | Speech | Ambulation | Dexterity | Emotion | Cognition | Pain or Discomfort |
|---|---|---|---|---|---|---|---|---|---|
| Control group | 0.92 | 0.97 | 0.99 | 0.98 | 0.99 | 0.99 | 0.98 | 0.98 | 0.99 |
| Marital status | | | | | | | | | |
| Married | 0.98 | 0.88 | 0.82 | 0.81 | 0.80 | 0.78 | 0.86 | 0.96 | 0.93 |
| Single | 0.97 | 0.77 | 0.82 | 0.76 | 0.84 | 0.79 | 0.84 | **0.86** | **0.83** |
| Level of education | | | | | | | | | |
| Less than secondary | **0.82** | 0.92 | 0.85 | 0.83 | 0.83 | 0.83 | 0.91 | **0.85** | 0.88 |
| Secondary | 0.93 | 0.93 | 0.90 | 0.86 | 0.89 | 0.87 | 0.95 | 0.94 | 0.93 |
| Other secondary | 0.98 | 0.97 | 0.98 | 0.98 | 0.97 | 0.97 | 0.96 | 0.96 | 0.97 |
| Postsecondary | 0.99 | 0.98 | 0.98 | 0.97 | 0.98 | 0.98 | 0.98 | 0.97 | 0.98 |
| Family income/year | | | | | | | | | |
| Less than $30,000 | **0.84** | **0.83** | **0.83** | **0.84** | **0.82** | **0.83** | **0.86** | **0.85** | **0.85** |
| $30,000–49,999 | **0.87** | 0.89 | **0.87** | **0.85** | **0.84** | **0.85** | 0.93 | 0.93 | 0.92 |
| $50,000–79,999 | 0.93 | 0.98 | 0.97 | 0.96 | 0.97 | 0.97 | 0.96 | 0.96 | 0.97 |
| $80,000 or over | 0.97 | 0.98 | 0.97 | 0.96 | 0.98 | 0.98 | 0.97 | 0.99 | 0.98 |
| Physical activity | | | | | | | | | |
| Active | **0.93** | 0.96 | 0.97 | 0.98 | 0.98 | 0.98 | 0.97 | 0.97 | 0.97 |
| Moderate | **0.92** | 0.92 | 0.87 | 0.85 | 0.85 | 0.86 | **0.91** | 0.94 | 0.89 |
| Inactive | 0.89 | 0.87 | 0.85 | 0.81 | 0.84 | 0.83 | **0.89** | **0.90** | 0.88 |
| BMI | | | | | | | | | |
| Less than 25.0 | 0.99 | 0.99 | 0.98 | 0.97 | 0.98 | 0.97 | 0.97 | 0.98 | 0.98 |
| 25.0–30.0 | **0.95** | 0.98 | 0.87 | 0.87 | 0.85 | 0.88 | 0.94 | **0.90** | 0.97 |
| 30.0+ | **0.91** | 0.90 | **0.82** | 0.81 | 0.82 | 0.83 | 0.93 | **0.89** | 0.90 |
| Cigarette smoking status | | | | | | | | | |
| Daily smoke | 0.93 | 0.90 | 0.86 | 0.82 | 0.87 | 0.83 | 0.89 | 0.92 | 0.86 |
| Occasional smoke | 0.96 | 0.95 | 0.93 | 0.85 | 0.88 | 0.85 | 0.91 | **0.94** | 0.94 |
| Former smoke | **0.92** | 0.94 | 0.89 | 0.89 | 0.90 | 0.88 | 0.94 | 0.97 | 0.93 |
| Never smoke | 0.98 | 0.99 | 0.98 | 0.97 | 0.97 | 0.98 | 0.99 | 0.99 | 0.99 |
| Alcohol drinking | | | | | | | | | |
| Yes | 0.88 | 0.93 | 0.96 | 0.94 | **0.92** | 0.94 | 0.96 | 0.96 | 0.92 |
| No | 0.97 | 0.94 | 0.99 | 0.96 | 0.97 | 0.97 | 0.97 | 0.99 | 0.99 |

[a] Adjusted for age and sex.

[b] Bold entries are statistically significant difference (0.03 for overall scores and 0.05 for single-attribute scores) and clinically important differences between 1994–1995 and 2002–2003.

than in 2002–2003. However, there were only few significant differences for physical inactivity in emotion and cognition. The scores of overweight or obesity in hearing, cognition, the scores of cigarette smoke in cognition, and the scores of heavy alcohol drinking in ambulation were also greater than 0.05 (Tables 3A and 3B). These results are similar to other studies that report that outcomes are correlated with age, sex, socioeconomic status, and personal habits, and that people with diabetes in primary health care have a markedly lower HRQOL compared to a standard population[19,20,21]. This can also explain that HRQOL for people with diabetes in 2002–2003 was worse than that in 1994–1995, perhaps due to a longer duration of diabetes, which could deteriorate complications due to diabetes.

We found that the largest deficits were among those with low family income (annual income less than $30,000 or from $30,000–$49,999). This has been recognized as one of the most important determinants of health care utility in the Canadian population[22,23]. Level of education was also found to be associated with HRQOL for individuals with diabetes, reflecting that it is an important determinant independent of its association with family income[23]. The fact that lower level of education (less than secondary) had a clinically important difference in HRQOL in this study showed that individuals with a higher level of education are likely to have a healthier lifestyle, including refraining from smoking, heavy alcohol drinking, with active physical activity and better access to healthy foods[22]. In diabetes patients, level of education can also influence the awareness of diabetes, skill of communication with healthcare providers, access to treatment choices, and ability to adhere to complex self-care regimens, which in turn can affect HRQOL[24].

Emotion was another source of heterogeneity where clinically important differences were observed between respondents with diabetes and those without. Respondents who rated themselves as “Unhappy/life is not worthwhile” or “Unhappy/little interest in life” reported HRQOL deficits very clinically important relative to those who rated themselves as “Happy and interested in life”. Stress is recognized as a determinant of health in general, but high levels of stress may be particularly problematic for individuals with diabetes, as stress is associated with poor glycemic control[25,26]. One study showed a statistically significant difference and an impact with a large effect size on every dimension in HRQOL effects between diabetes patients with and without depression in the physical and mental component summaries of the Short Form (SF-36) in HRQOL questionnaire[27]. This means that emotion and depression are important components in measuring HRQOL for diabetic patients. Another prospective study showed that children born to mothers in the lowest socioeconomic group were more likely to be impaired in their mental/emotional health later in childhood[28].

HRQOL was associated with marital status of respondents reported with diabetes. Single respondents (including separated, divorced, or widowed) generally experienced lower levels of quality of life compared to those who were married ($p < 0.01$). Children from unmarried and divorced mothers were significantly below the average with respect to quality of life[29]. HRQOL measures are sensitive to clinical characteristics, such as frequency and severity of complications[30]. For those people with diabetes who are married, HRQOL was better among the insulin-treated than noninsulin-treated[31].

### Limitations

Due to limitation of data design and data collection, we could only compare the differences between the study group and control group. We were not able to compare the potential changes in HRQOL over time in persons with diabetes between 1994–1995 and 2002–2003, and present the 1994–1995 and 2002–2003 HUI data in the 401 subjects who started in the original cohort separate from all 587 subjects at 2003.

The current study relied on self-reported diabetes status. Manuel and Schultz reported that self-reported diabetes appears to have considerable underreporting compared to physician-diagnosed diabetes in the NPHS[32]. The sensitivity is only 47% in comparison with the physician-diagnosed diabetes, being used as a "gold-standard"[32]. Furthermore, they reported large differences in HUI3 scores between people who have physician-diagnosed diabetes and self-reported diabetes. A further limitation of this study was an inability to differentiate between type 1 and type 2 diabetes. However, fewer than 10% of cases of diabetes among those age 20 years or older are type 1[33].

### CONCLUSIONS

The results of this study showed statistically significant and clinically important differences in the HRQOL and the demographics, lifestyle, and socioeconomic status of individuals with diabetes compared to those without. The HRQOL for people with diabetes were worse in 2002–2003 than in 1994–1995 when we compared the overall HUI3 scores and the eight attribute utility scores of those with diabetes to the scores of those without diabetes in the NPHS longitudinal data. The HUI3 system is considered a good indicator in measuring the functional health status and HRQOL for patients with diabetes compared to those without.
